# A Case of Delayed Hepatic Injury Associated with Teriflunomide Use as Assessed for Causality Using the Updated RUCAM

**DOI:** 10.1155/2022/6331923

**Published:** 2022-06-15

**Authors:** Riana Wurzburger

**Affiliations:** Department of Internal Medicine, University of New Mexico Hospital, UNM School of Medicine MSC08 47201, University of New Mexico, Albuquerque, NM 87131, USA

## Abstract

Teriflunomide is a pyrimidine synthesis inhibitor used in the treatment of multiple sclerosis that has in rare instances been associated with liver toxicity, though there are few documented cases. Here, we report a case of probable teriflunomide-induced liver injury as assessed for causality using the updated RUCAM. The liver injury occurred approximately nine months after teriflunomide initiation and improved with discontinuation of the drug and treatment with cholestyramine.

## 1. Introduction

Teriflunomide is an FDA-approved immunomodulator used for treatment of relapsing-remitting forms of multiple sclerosis [1]. It is the active metabolite of leflunomide, a disease modifying antirheumatic drug that has been frequently associated with liver enzyme elevation, and rarely with severe liver injury [2]. In controlled trials of teriflunomide, the most common hepatic adverse event was a transient elevation in liver function tests, but there was also one case of severe liver injury in which a drug effect could not be excluded [2, 3]. Given this and the known risk of liver injury with parent-drug leflunomide, teriflunomide carries a black box warning for hepatotoxicity [3]. There are, however, few documented case reports of teriflunomide-induced liver injury [4–6]. Here, we report a case of probable drug-induced liver injury (DILI) attributed to teriflunomide use.

## 2. Case Presentation

A 60-year-old woman with relapsing multiple sclerosis presented from her long-term care facility with altered mental status, fatigue, anorexia, and jaundice. She had been started on teriflunomide therapy (14 mg daily) nine months prior, at which point liver tests (LTs) were normal.

On arrival, she was afebrile, tachycardic, and normotensive, without an oxygen requirement. Labs were significant for aspartate transaminase (AST) of 1,499 U/L, alanine aminotransferase (ALT) of 777 U/L, alkaline phosphatase (AP) of 478 U/L, and total bilirubin (Tbil) of 2.5 mg/dL, without significant abnormalities on complete blood count or chemistry panel. The INR was 1.20. Lactate and lipase were normal, acetaminophen level was <2 ug/ml, and COVID-19 and influenza A and B were negative. Initial CT and abdominal ultrasound showed nonspecific hepatic echogenicity, patent hepatic vessels, and no abnormalities in the biliary system. Teriflunomide was discontinued immediately upon admission given the black box warning for hepatotoxicity, and she was admitted for workup. Hepatitis and herpes serologies were negative. Antinuclear antibody, liver-kidney microsomal antibody, immunoglobulin G levels, alpha-1 antitrypsin, serum protein electrophoresis, and iron levels were normal. Rheumatologic workup was notable for a positive smooth muscle antibody with a titer of 1 : 40, which was thought to be clinically insignificant. Ferritin was significantly elevated at 28,754 ng/ml; however, MRI with iron quantification showed no evidence of iron overload. Hemochromatosis gene testing revealed heterozygosity for the H63D mutation, a genotype that has not been clearly associated with symptoms of hereditary hemochromatosis [7]. In combination with the MRI and other lab results, this was not thought to be clinically significant. Over the course of admission, AST and ALT trended down, but AP, Tbil, and INR continued to rise. ERCP with EUS and liver biopsy were performed on hospital day 5. Biopsy was most consistent with drug-induced injury, with a minor component of steatohepatitis. Teriflunomide was the suspected culprit drug, and the patient was treated with cholestyramine 4 grams every 6 hours for accelerated elimination starting on hospital day 7. LTs dropped consistently across all lab markers by hospital day 10 (Table 1). Teriflunomide was discontinued indefinitely at the time of discharge back to her long-term care facility. At one month follow-up, LTs were markedly improved (Table 1).

## 3. Discussion

While hepatotoxicity is listed as a potential complication of treatment with teriflunomide, there are few available case reports that demonstrate this effect [4–6]. Causality for teriflunomide-induced DILI was assessed in this case using the updated RUCAM score, a well-established tool used to determine the likelihood that a hepatic injury is due to a specific drug [8]. The RUCAM scale requires calculation of the *R* factor (the pattern of liver injury) and then assigns points for 7 different components of the history and laboratory findings to create a likelihood score for DILI (≤0, excluded; 1-2, unlikely; 3–5, possible; 6–8, probable; and ≥9, highly probable) [8].

In this case, the calculated *R* factor was 3.6, consistent with a mixed liver injury. A review of available case reports on teriflunomide and leflunomide-associated hepatotoxicity suggests that hepatocellular injury is the most common, though mixed liver injury has been documented as well [5, 9]. The updated RUCAM score was calculated as 6 for this case, meaning teriflunomide-induced DILI was probable. While the patient was on omeprazole and mirtazapine, each of these was a long-term (>5 year) medication without associated LT abnormalities on routine monitoring and as such were considered incompatible with time to onset of DILI. In the search for alternative causes, several virologic studies were not completed at the time of admission (CMV, EBV, VZV, and HEV), and so acute CMV, EBV, VZV, or hepatitis E infections could not be ruled out. Even without excluding these viruses, hepatic injury from teriflunomide was still deemed probable by the RUCAM score.

The timing of liver injury is an interesting point in this case. The RUCAM score awards more points for injury that occurs within 5–90 days of medication initiation, and in the teriflunomide drug trial, DILI was more likely in the first weeks to months of treatment [2, 3]. Our patient developed liver injury 9 months after initiation of the medication, suggesting that hepatotoxicity can occur at various points in treatment. This emphasizes the importance of regular LT monitoring with teriflunomide therapy. Once liver injury (or any toxicity) is suspected, it is recommended to use cholestyramine or activated charcoal for accelerated elimination [3]. In our case, there was a delay in starting cholestyramine; however, initiation was associated with the onset of LT improvement (Table 1), as seen in other case reports [5]. This highlights the importance of providing treatment for accelerated elimination if teriflunomide-induced DILI is suspected.

This case adds to the limited number of reports demonstrating the potential for liver injury with teriflunomide. More data and case reports will be helpful in determining the frequency and timing of hepatic toxicity in the case of teriflunomide-induced liver injury.

## Figures and Tables

**Table 1:** Patient LTs and INR over the course of admission.

	AST (units/L)	ALT (units/L)	AP (units/L)	Tbil mg/dL	INR
Reference range	6–58 u/L	14–67 u/L	38–150 u/L	0.3–1.2 mg/dL	1
Admission	1,499	777	478	2.5	1.2
Hospital day 1	842	583	442	3.2	
Hospital day 2	527	442	595	4.4	
Hospital day 3	403	392	643	5.4	
Hospital day 4	368	341	759	6.5	1.37
Hospital day 5	404	361	929	8.6	
Hospital day 6	449	402	972	9.7	1.66
Hospital day 7^*∗*^	366	357	894	10	1.97
Hospital day 8	289	323	892	10.9	1.73
Hospital day 9	259	302	919	10.7	1.64
Hospital day 10	232	278	891	9.2	
Discharge	170	232	769	7.8	
One month follow-up	33	21	355	0.9	1.26

^*∗*^Initiation of cholestyramine.

## Data Availability

The data used to support the findings of this case report are included within the article.
